# Managing discordance between HbA_1c_ and glucose management indicator

**DOI:** 10.1111/dme.70023

**Published:** 2025-03-23

**Authors:** Erna Lenters‐Westra, Marion Fokkert, Eric S. Kilpatrick, Erwin Schleicher, Scott Pilla, Emma English, Peter van Dijk

**Affiliations:** ^1^ Department of Clinical Chemistry Isala Zwolle the Netherlands; ^2^ European Reference Laboratory for Glycohemoglobin the Netherlands; ^3^ Department of Clinical Biochemistry Manchester Foundation Trust Manchester UK; ^4^ Department for Diagnostic Laboratory Medicine, Institute for Clinical Chemistry and Pathobiochemistry University Hospital Tuebingen Tuebingen Germany; ^5^ Division of General Internal Medicine, Department of Medicine Johns Hopkins University School of Medicine Baltimore Maryland USA; ^6^ University of Cambridge Madingley Hall UK; ^7^ Department of Internal Medicine, Diabetes Centre Isala Zwolle the Netherlands; ^8^ Department of Endocrinology University of Groningen, University Medical Center Groningen the Netherlands

**Keywords:** diabetes, discordance, GMI, HbA_1c_

## Abstract

**Aims:**

The assessment of haemoglobin A1c (HbA_1c_) continues to play an essential role in diabetes care; however, major advances in new technologies widen the armament available to clinicians to further refine treatment for their patients. Whilst HbA_1c_ remains a critical glycaemic marker, advances in technologies such as Continuous Glucose Monitoring (CGM) now offer real‐time glucose monitoring, allowing a more instant assessment of glycaemic control. Discrepancies between laboratory‐measured HbA_1c_ and Glucose Management Indicator (GMI) values are a significant clinical issue. In this article, we present a checklist of potential sources of error for both GMI and HbA_1c_ values and provide suggestions to mitigate these sources in order to continue to improve diabetes care.

**Methods:**

We identified key literature pertaining to GMI measurement, HbA_1c_ measurement, and potential factors of discordance between the two. Using these sources, we explore the potential factors leading to discordance and how to mitigate these when found.

**Results:**

We have constructed a quick reference checklist covering the main sources of discordance between HbA_1c_ and GMI, with accompanying narrative text for more detailed discussion. Discordance can arise due to various factors, including CGM accuracy, sensor calibration, red blood cell turnover and other physiological conditions.

**Conclusions:**

GMI will likely continue to be used in the upcoming years by both persons with diabetes and their health care providers, and so it is important for users of CGM devices to be equipped with the knowledge to understand the potential causes of discordance between GMI and HbA_1c_ values.

**What‘s new?**

- The introduction of the glucose managment indicator (GMI) is a great tool for assessing a person’s glycaemic status however values between GMI and HbA_1c_ may be discordant.
- We have developed a checklist of potential sources of error for both GMI and HbA_1c_ values, with suggested steps to mitigate or reduce sources of discordance.
- This will lead to a better understanding and management of the discordance between GMI and HbA_1c_ and enable improved treatment decisions which lead to improved outcomes.

## 1 INTRODUCTION

Assessment of HbA_1c_ has been a cornerstone of diabetes management for nearly half a century. Landmark studies like the Diabetes Control Complication Trial (DCCT) and the United Kingdom Prospective Diabetes Study (UKPDS) have unequivocally shown the relationship between HbA_1c_ and the development of long‐term complications of diabetes.^1^, ^2^, ^3^ These seminal trials resulted in target values for HbA_1c_ in the management of diabetes that continue to be in use today, and current practice guidelines emphasise an individualised approach to setting HbA_1c_ target values, taking into consideration factors such as coexisting chronic illnesses, age and risk of hypoglycaemia.^4^

The options for HbA_1c_ measurement have grown significantly over recent decades, and there is a baffling array of analytical instruments and measurement formats available. All methods should be standardised and traceable to the International Federation of Clinical Chemistry and Laboratory Medicine (IFCC) Primary Reference Measurement Procedure (PRMP) and harmonised with the method used in the DCCT and UKPDS via the National Glycohemoglobin Standardization Program (NGSP).^5^, ^6^ Details of methods that have evidenced this are available from the respective organisations.^7^, ^8^

Whilst HbA_1c_ is strongly correlated with the risk of long‐term micro‐ and macrovascular diabetes complications, it does not provide information on short‐term fluctuations in glucose levels, overall glycaemic variability or hypoglycaemia. Interest in Continuous Glucose Monitoring (CGM) has been growing dramatically in research and clinical use. CGM allows glucose levels to be assessed close to real time in the patient setting, with the additional option for pre‐set (predictive) alarms. Typically, transcutaneous enzymatic (mostly glucose oxidase) sensors measure glucose every few (~5–15) minutes, 24 h a day during the lifespan of the sensor (~7–14 days) after which the sensor needs to be replaced. Systems that place a larger sensor under the skin have a longer lifespan of up to 180 days and use fluorescence to measure glucose. It should be noted that CGM devices measure interstitial glucose, not blood glucose; the devices use an algorithm to convert the measured interstitial glucose to an equivalent blood glucose value. In recent years, CGM technology has evolved, becoming smaller, more accurate and easier to use, and has led to improvements in glycaemic control and quality of life among persons with diabetes.^9^ Depending on health care settings, reimbursement options and the (sub)populations involved, the uptake of CGM in persons with diabetes has increased dramatically. It is estimated that between 30 and 50% of privately or publicly insured persons in the United States with type 1 diabetes on intensive insulin therapy use CGM.^10^ Among persons with non‐insulin‐based therapy (mostly persons with type 2 diabetes), the uptake of CGM is lower; yet it is expected to increase in the upcoming decades as it offers valuable feedback on the impact of lifestyle (diet and exercise) on glucose levels.^11^

The huge number of data points that are generated from CGM has led to a plethora of different approaches in interpreting and applying that data for diabetes management. The ambulatory glucose profile (AGP) is the whole of the data generated, and this is further broken down to metrics with target values such as: number of days CGM device is worn, percentage of time CGM device is active, mean glucose, time in range (TIR), time above range (TAR), time below range (TBR) and glucose variability.^12^

Whilst these different metrics all give the user information on different aspects of glycaemia, the metric of interest in this article is the Glucose Management Indicator (GMI) which is a measure that converts the mean glucose values, obtained from CGM, into an estimate of HbA_1c_.

## 2 DEFINING GMI

GMI was previously described as estimated A_1c_, with the name (falsely) suggesting that the estimated HbA_1c_ would give a similar result as HbA_1c_ measured in a laboratory or with point‐of‐care (POC) devices. To avoid confusion between the derived estimated A_1c_ and a laboratory (or POC) measured HbA_1c_, the name was changed to GMI.^13^ The A_1c_‐Derived Average Glucose (ADAG) study in 2006–2007 generated an equation used to estimate mean glucose based on HbA_1c_.^14^ The GMI equation is effectively the reverse of this and uses a mean glucose value to approximate an HbA_1c_ value (GMI).^15^ Questions were raised by Norris and Lang as to the validity of the equation and they suggested a revision of the ADAG‐ and DCCT‐based mean average glucose to estimate A_1c_.^16^ Since the ADAG study, many more formulas and kinetic models have been introduced to estimate HbA_1c_.^13^, ^17^ However, it is often unclear which equations are used by different CGM devices to estimate HbA_1c_.

There are often discrepancies between measured HbA_1c_ and GMI derived from CGM data.^13^, ^15^ These inconsistencies can cause confusion among health care providers and persons with diabetes. An expert recently suggested that GMI should not be reported as it does not always accurately reflect HbA_1c_ values and instead rely on CGM mean glucose.^15^ A counter response argued that most health care providers and persons with diabetes do not have the contextual experience or knowledge of how to interpret mean glucose and how much of a change in mean glucose is clinically meaningful, whereas HbA_1c_ is better understood.^18^ Given the increase in CGM users and the increasing tendency towards more remote diabetes care, we believe that persons with diabetes and health care providers will continue to use GMI, irrespective of any merits or demerits. Therefore, it is important to increase awareness of potential discordance between GMI and HbA_1c_ values, acknowledging that both metrics have underlying sources of error, understanding and identifying potential causes, and being able to investigate and mitigate these causes, where possible.

## 3 DEFINING DISCORDANCE BETWEEN GMI AND HbA_1c_

In 2021, Perlman et al. compared HbA_1c_ with GMI and found that only 11% of the participants (*n* = 641) had an absolute HbA_1c_–GMI discordance of less than 1 mmol/mol (0.1%), whilst 50% and 22% had differences ≥5 mmol/mol (0.5%) and ≥11 mmol/mol (1%) respectively.^19^ In another study by Bergenstal et al., it was shown that among 528 individuals, 28% had a GMI that differed from the laboratory‐measured HbA_1c_ by ≥5 mmol/mol (0.5%), 19% by ≥7 mmol/mol (0.6%), 12% by ≥8 mmol/mol (0.7%) and 8% by ≥9 mmol/mol (0.8%).^13^ The discordance between GMI and HbA_1c_ appears to increase with increasing HbA_1c_ concentration, which may be a reflection of greater glucose variability in people with higher levels of glycaemia. Recent findings by Sterner Issaksom et al. (2024) further elucidate this discordance.^20^ Their study demonstrated that for a given mean glucose (MG) and TIR, the HbA_1c_ of 10% of individuals deviated by more than 9 mmol/mol (0.8%) from their estimated HbA_1c_ based on the overall association between MG and TIR with HbA_1c_. At a given TIR, each 1% increase in TBR was related to a 0.6 mmol/mol (0.05%) lower HbA_1c_, and each 2% increase in TAR level 2 was related to a 0.4 mmol/mol (0.04%) higher HbA_1c_. This significant deviation underscores the variability and potential discordance between these measures, particularly in individuals with type 1 diabetes.

There is no established definition of discordance between GMI and HbA_1c_. A difference between two consecutive HbA_1c_ values of 5.5 mmol/mol (0.5%) has been used as a clinically relevant threshold to warrant making adjustments to therapy, and this has been translated into studies looking at discordance between GMI and HbA_1c_ values.^13^, ^15^ Although the studies above show quite different rates of discordance, they do indicate that at 5.5 mmol/mol (0.5%) the number of cases of discordance is likely to be significant.

The analytical performance that can be achieved with laboratory HbA_1c_ methods is evidenced through External Quality Assessment (EQA) schemes. For a sample with a true value of 48 mmol/mol (6.5%) one laboratory might report 43 mmol/mol (6.1%) and another 52 mmol/mol (6.9%); these values would pass EQA criteria but could result in a difference of up to 9 mmol/mol (0.8%) between different laboratories. Therefore, setting the limit for discordance at a level of 9 mmol/mol (0.8%) takes the analytical performance of HbA_1c_ methods into consideration and is a pragmatic starting point for both clinicians and persons with diabetes to start a conversation around the investigation of the discordance.^21^

There is still a paucity of data from which to draw robust conclusions; however, based on the studies described and EQA performance data for HbA_1c_, we suggest that a difference of 9 mmol/mol (0.8%) between HbA_1c_ and GMI is discordant and should warrant further investigation. Whilst a visit‐to‐visit difference of >5.5 mmol/mol (0.5%) is often considered a clinical decision point for change in therapy, there are still many HbA_1c_ instruments where the analytical uncertainty in measurement could result in such a difference; hence, the wider range of 9 mmol/mol (0.8%) is considered more meaningful in the context of discordance between laboratory‐measured HbA_1c_ and GMI. Clinical experience of individual cases should always be taken into account when determining whether discordance is present, especially if a consistent difference in values is observed.

## 4 INVESTIGATING SOURCES OF DISCORDANCE BETWEEN GMI AND HbA_1c_

Tables 1 and 2 provide a checklist of potential sources of error for both GMI and HbA_1c_ values and provide suggestions to mitigate these sources in order to identify and reduce discordance. Further narrative detail is provided below.

**TABLE 1:** Checklist of potential sources of error in GMI.

Potential sources of error in GMI	Impact on GMI (relative to true glycaemic level)	Recommended Action
*Data collection*		
CGM data available for <14 days?	GMI ↑↓	Use full 14‐day data with >70% data coverage
Less than 70% of the representative sensor data collected during the Ambulatory Glucose Profile (AGP) period?	GMI ↑↓	
*Change in equilibrium of glucose in interstitial fluid, blood and the cells*		
Episodes of (critical) illness (shock, oedema, hypotension, ketoacidosis)	GMI ↑↓	Re‐collect data from the next 14 days
Inflammation at the CGM insertion site	GMI ↓	Replace CGM
CGM sensor not sufficiently immobilised	GMI ↓	Replace CGM
Person with diabetes sleeps on the sensor	GMI ↓	Replace CGM (another insertion place)
CGM sensor is encapsulated (for subcutaneous devices)	GMI ↓	Replace CGM
*Accuracy of the CGM* measurements		
CGM was used under extreme exercise conditions	GMI ↑↓	Rely on HbA_1c_ Check glucose with BGM
CGM requiring calibration was not calibrated with BGM	GMI ↑↓	Rely on HbA_1c_ Ensure proper CGM calibration Consider changing to factory‐calibrated CGM
CGM requiring calibration was not calibrated in steady glycaemic state	GMI ↑↓	Rely on HbA_1c_ Calibrate in a steady glycaemic state as per manufacturer's instructions Consider changing to factory‐calibrated CGM
*Impact of calculations used to calculate GMI values*		
GMI values may be more discordant at low and high values of HbA_1c_ with the increases in GMI relative to HbA1c at the low levels and vice versa.	GMI ↑↓	Rely on HbA_1c_
*Drug interferences affecting CGM* values		
The CGM user is taking a substance interfering with their CGM device (see Table S1)	GMI ↓↑	Rely on HbA_1c_ Check glucose with BGM Discontinue interfering substance if possible, or change CGM device

**TABLE 2:** Checklist with potential sources of error in HbA_1c_.

Potential sources of error in HbA_1c_	Impact on HbA_1c_ (relative to true glycaemic level)	Recommended Action/Comment
*Data collection*		
HbA_1c_ was not measured in the same period as CGM wear	HbA_1c_ ↑↓	Re‐collect data during the same time period
*Factors that affect the interpretation of HbA*_*1c*_		
Recent (in prior 14 days) blood transfusion	HbA_1c_ ↓	Rely on CGM metrics until evidence of stable RBC population^a^
Recent considerable blood loss	HbA_1c_ ↓	Rely on CGM metrics until evidence of stable RBC population^a^
Active haemolysis	HbA_1c_ ↓	Rely on CGM metrics until evidence of stable RBC population^a^ Consider measuring LDH, haptoglobin and reticulocytes if degree of haemolysis is unclear
Chronic kidney disease stage 4 or ESRD	HbA_1c_ ↓	Rely on CGM metrics
Pregnancy	HbA_1c_ ↑↓	Rely on CGM metrics
Anaemia (due to iron deficiency or other cause)	HbA_1c_ ↑↓	Rely on CGM metrics until anaemia has been corrected^a^ Consider measuring full blood count and ferritin if degree of anaemia is unclear
Recent correction of iron deficiency anaemia	HbA_1c_ ↓	Rely on CGM metrics until evidence of stable RBC population^a^
Recent (<14 days) substantial increase in hyperglycaemia due to, for example, subscription of corticosteroids or non‐adherence to medications	HbA_1c_ ↓	Rely on CGM metrics
Recent (<14 days) substantial decrease in hyperglycaemia due to, for example, additional glucose‐lowering medication, carbohydrate‐reduced diet or intensive exercise regimen	HbA_1c_ ↑	Rely on CGM metrics
*Factors that interfere with HbA*_*1c*_ *measurement*		
Heterozygous Hb variant causing interference with laboratory assay	HbA_1c_ ↑↓	See: https://ngsp.org/factors.asp. Consult Laboratory Health Professional
Homozygous Hb variant causing interference with laboratory assay and interpretation of HbA_1c_	HbA_1c_ ↑↓	Rely on CGM metrics, do not report HbA_1c_
*Drug interferences affecting HbA*_*1c*_		
Person is taking any of the following drugs: dapsone, ribavrin, antiretroviral therapy, sulphasalazine, hydroxyurea or any drug that stimulates erythropoiesis	HbA_1c_ ↓	Rely on CGM metrics

^a^ Evidence of stable RBC population may be assessed using markers such as RDW.

## 5 POTENTIAL SOURCES OF ERROR IN GMI (RELATIVE TO TRUE GLYCAEMIC LEVEL)

### 5.1 Data collection period

Current guidelines recommend 14 days of CGM data with more than 70% of the representative sensor data collected during this time. Bailey et al. compared the GMI calculated using 14 days of CGM data with GMI calculated using <14 days and concluded that 10–14 days of CGM data are preferred, but that for most persons with diabetes a satisfactory estimate of HbA_1c_ can be obtained with 7 days of data.^22^ However, to minimise this as a potential source of discordance, we recommend using 14 days with >70% of the representative sensor data collected during the AGP period. A case study to illustrate the value of using data over the longer period is provided in File S1.

### 5.2 Equilibrium between glucose in interstitial fluid, blood and the cells

As glucose is measured in the interstitial fluid with CGM and not in blood, any conditions affecting the equilibrium between glucose in the interstitial fluid, blood and the cells will have an impact on the glucose concentrations measured with CGM. If that condition is for a prolonged period, it will ultimately influence the GMI. Examples of some of these conditions are:

1. Physiological effects related to (critical) illness or post‐surgery (shock, oedema, hypotension etc.): can cause temporarily higher glucose concentrations and may lead eventually to a higher GMI if the length of time of hyperglycaemia is significant.
2. Inflammatory reaction at the sensor insertion site: reduces the local glucose concentration as glucose is used by inflammatory cells and, if sustained, lowers GMI^23^
3. When a sensor is not properly attached to the ski, it causes mechanical stress/friction between the sensor and skin, which may lead to lower glucose concentrations and eventually to a lower GMI.
4. Compression of the sensor (for instance during sleep): may lead to reduced diffusion of glucose at the sensor site, which in turn may lead to lower glucose concentrations and, over time, to a lower GMI (please see File S1 for an example case)

### 5.3 Performance of CGM devices in extreme physiological conditions

In extreme exercise conditions, interstitial glucose readings with CGM are less accurate compared with capillary measurements, especially at low glucose values.^24^

### 5.4 Issues surrounding standardisation of CGM values

Although a consensus was formed around standardising the reporting format of summarised CGM data, as a description of the AGP, the standardisation of CGM glucose measurements themselves has not yet reached this point.^25^, ^26^ Analytical performance of CGM systems differs not only between manufacturers but also between individual sensors of the same system and sometimes even within the same person.^27^, ^28^

Several issues arise with glucose measurements in the interstitial compartment, which has a different matrix and different flow dynamics, including differences in rates of change of glucose. This means changes in interstitial fluid glucose often lag behind those of blood glucose by up to 10–15 min.^26^ The IFCC Scientific Division Working Group on continuous glucose monitoring was established with the aim to standardise CGM to a higher‐order standard or a Primary Reference Measurement Procedure (PRMP) and to provide recommendations for reporting the general study design, CGM system use, the comparator measurement approach, testing procedures and data analysis/statistical performance evaluation.^29^ It is no surprise that standardising CGM is not an easy task, as glucose is not a stable parameter, and different CGM and different statistical approaches can lead to different outcomes and thus ultimately influence the discordance between GMI and HbA_1c_.

### 5.5 Impact of using different equations to calculate GMI

As noted, the most authoritative equation between mean blood glucose and estimated HbA_1c_ was established in the ADAG study in 2006–2007.^14^ New conversion formulas were established in 2017 by Beck et al. and in 2018 by Heinemann, of which the latter was validated with CGM data from the HypoDE study.^30^, ^31^ Subsequently, these two equations were combined for a greater precision by Bergenstal et al.^13^ As well as different equations being used to link glucose to HbA_1c_, different mathematical models have also been developed. Valenzano et al. developed a more precise mathematical model to calculate GMI derived from CGM data.^17^ However, it is often not clear which equation is used by each manufacturer, which alone may lead to confusion, with different equations leading to different GMI results when applied to the same glucose profile. A further complicating factor is the way in which third‐party programs calculate CGM‐derived metrics; Karakus et al. showed that the use of different programs for the analysis of the AGP resulted in statistically different values for TIR in TAR in some cases.^32^ Although data for GMI was not included in the study, it raises the question as to how much variability could arise through the use of these programs to calculate GMI.

### 5.6 Impact of manual calibration on CGM values

There are CGM systems that require frequent user manual calibration, with capillary blood glucose measurements (handheld Blood Glucose Meter (BGM)); others are factory‐calibrated and do not need user input. There are several sources of error with the use of manual calibration: user error in inputting the values, user error in the timing of calibration and the accuracy of the BGM device used to provide the calibration values. Each of these can introduce significant differences between the reported glucose value and the actual glucose value and thus ultimately affect the GMI.^33^, ^34^

Incorrect timing of the calibration (during a meal or insulin bolus) when the discrepancy in glucose between interstitial and blood will be greatest will result in the CGM glucose values being misaligned with the blood glucose value, which will ultimately lead to a GMI incorrectly lower or higher than the HbA_1c_.

### 5.7 Interference of drugs

Many drugs, including common ones such as acetaminophen (paracetamol), may interfere with glucose measurement, so the manufacturer's instructions should be followed when interpreting glucose and/or GMI values in their presence.^35^ Table S1 provides an overview of potential sources of drug interactions. However, for the most current information, it is recommended to check the latest user manual and manufacturer's updates that may be available online. The level of interference depends on the amount of drug active in the body and the clearance of the drug. A pragmatic approach to investigate/detect (new) potentially interfering drugs is to perform a 7‐point (fingerstick) glucose measurement with a BGM and compare it with CGM glucose, both with and, if possible, without the drug present in the body. If the discrepancy in glucose measured with BGM compared to glucose measured with CGM during drug use is greater than without drug use, this may indicate interference.

## 6 POTENTIAL SOURCES OF ERROR IN HbA_1c_

There is a distinction between factors that interfere with the measurement of HbA_1c_ and those that interfere with its interpretation. In the first instance, the method yields an analytically incorrect HbA_1c_ result, whereas in the second instance, the HbA_1c_ result is analytically accurate but does not correlate with a person's glucose status.

## 7 FACTORS THAT AFFECT THE INTERPRETATION OF HbA_1c_

### 7.1 Differences in the life span of red blood cells (RBC) between individuals

Although it is commonly asserted that the lifespan of the red blood cell is approximately 120 days, there is a wide variance around this value, varying between 70 and 140 days.^36^ Between individuals, the lifespan varies by approximately ±15%.^37^ As HbA_1c_ formation is dependent on time, these differences result in different HbA_1c_ values independent of recent glucose exposure.

### 7.2 Turnover of Red Blood Cells (RBC)

Conditions, some of which are treatable, that affect the turnover of RBC and consequently RBC lifespan, may affect HbA_1c_ formation. A higher turnover of cells leads to a lower rate of HbA_1c_ formation and thus a lower value (often termed ‘falsely low’ as it does not represent the true glucose exposure) and conversely, any condition that decreases red cell turnover will result in a longer time of exposure of the cell to glucose and thus more HbA_1c_ formation. Some guidelines suggest measuring alternative markers, such as fructosamine and glycated albumin, in these circumstances. In people with chronic kidney disease (CKD), hypoalbuminaemia due to protein losses in the urine, malnutrition or peritoneal dialysis is a common condition, and therefore, glycated albumin and fructosamine may not accurately reflect actual blood glucose.^38^ A recent study showed the limitations of glycated albumin standardisation when applied to the assessment of persons with diabetes.^39^

Conditions that affect the rate of turnover include:

1. Acute blood loss: increased red cell turnover leading to falsely low HbA_1c_.
2. Haemolysis, for example, due to drugs (dapsone or penicillin derivates), irregular RBC antibodies or secondary autoimmune reactions: increased red cell turnover leading to falsely low HbA_1c_.^40^, ^41^, ^42^ Elevated LDH and reduced haptoglobin are markers of haemolysis, and elevated reticulocytes are an indication of high turnover of RBC.
3. Iron deficiency anaemia (IDA): HbA_1c_ may be falsely elevated in mild to moderate IDA but may be lower in severe IDA or with the correction of iron deficiency.^43^
4. Non‐iron deficiency anaemia: HbA_1c_ may be falsely low due to a non‐iron deficiency anaemia.^43^
5. Chronic kidney disease (CKD) and end‐stage renal disease (ESRD): ESRD presents a complicated mixed picture; there may be iron deficiency which may increase HbA_1c_, but conversely, this is affected by the use of erythropoietin which will stimulate RBC turnover and increase reticulocyte formation.^38^ In addition, the reduction of renal function may reduce the RBC lifespan before overt ESRD.^44^ Overall, HbA_1c_ values tend to be lower than expected in those with ESRD and in persons with more severe CKD (CKD 4). The guidelines for diabetes management in chronic kidney disease recommend not to use HbA_1c_ when the estimated glomerular filtration rate (eGFR) is <30 mL/min/1.73 m^2^.^38^
6. Pregnancy: HbA_1c_ is reduced in the second trimester but is increased in the third trimester.^45^
7. Homozygous and combined Hb variants (HbSS, HbCC, HbSC etc.): HbA_1c_ should not be reported if the Hb variant is homozygous, given the pathological processes, including anaemia, increased red cell turnover and transfusion requirements, that adversely impact HbA_1c_ as a marker of long‐term glycaemic control.^46^

When any condition that could potentially affect RBC turnover is considered, it is important to first correctly identify the issue, then, if possible, treat and resolve the underlying issue before relying on HbA_1c_. This may take 6 months, and RBC indices such as red cell distribution width (RDW) can offer a marker of stabilisation of the RBC population.

## 8 FACTORS THAT INTERFERE WITH HbA_1c_ MEASUREMENT

### 8.1 Interference from Hb variants

Some Hb variants interfere with HbA_1c_ measurement.^47^ There is, unfortunately, no fixed pattern of interference, and this needs to be investigated with a case‐by‐case approach. Both laboratory and POC devices may be affected. Most of the HbA_1c_ methods used in a laboratory setting do not have interference from the common Hb variants (HbAS, HbAC, HbAD and HbAE); however, this is not clear for many POC devices. Health care providers should suspect the presence of an Hb variant when an HbA_1c_ result is unexpectedly significantly different from a previous HbA_1c_ result or when GMI and other CGM metrics are consistently different than expected when compared to HbA_1c_. Potential causes such as a recent change in laboratory or POC method have been ruled out.

Many Hb variants and high fetal Hb (HbF) concentrations can be identified in the chromatogram of cation exchange HPLC methods (when in variant mode rather than the standard or fast mode) and capillary electrophoresis methods but not in immunoassays, enzymatic and most of the POC methods, as these methods only report a result. Therefore, caution should be exercised before ruling out an Hb variant as a potential confounding factor. Communication between the health care provider and the laboratory health professional where the HbA_1c_ was analysed is essential to query and identify a potential Hb variant. The following websites can be useful:

1. the NGSP website to check whether the HbA_1c_ method has an interference from an Hb variant^47^ and
2. the ithanet website for the prevalence of Hb variants worldwide.^48^

## 9 GENERAL CONCLUSIONS

The evolution in both HbA_1c_ measurement and CGM technologies is transforming diabetes management with a shift towards more personalised and real‐time approaches to therapeutic interventions. It is important to understand the purpose and value of these tests so that they can be used safely and effectively in patient care. CGM is a tool for providing real‐time management of diabetes, and HbA_1c_ is a monitoring tool used to demonstrate the efficacy of that management. The rate of change of GMI may prove useful as a tool to assess more immediately the efficacy of therapeutic approaches. Both markers can provide valuable insight into patient care and are complementary indicators. However, health professionals should be cognisant of the role they expect these markers to play in patient management before their use.

We propose that an absolute difference of 9 mmol/mol (0.8%) between GMI and HbA_1c_ is considered a significant discordance, warranting further investigation. The checklist presented in this article helps persons with diabetes and health care providers identify the possible causes for discordance and proposes actions to mitigate these where possible.

## AUTHOR CONTRIBUTIONS

E.L‐W. and E.E. researched data, contributed to discussion and wrote the first draft of the manuscript. M.F., S.P. and P.v.D. contributed to discussion, reviewed and edited the manuscript. E.S.K. and E.S. reviewed and edited the manuscript. All authors approved the final version of the manuscript. E.L‐W. and E.E. act as guarantors for this manuscript.

## FUNDING INFORMATION

No funding was recieved for this article.

## CONFLICT OF INTEREST STATEMENT

All authors have declared that they have no potential conflicts relevant to this article.

## Supporting information


File S1.

Table S1.
