# Supplementary material for: Managing discordance between HbA1c and glucose management indicator
Source: Diabet Med. 2025 Mar 23;42(6):e70023. doi: 10.1111/dme.70023 (PMC12080991; doi:10.1111/dme.70023)
Supplement: Supplementary file 1 — File S1. [file DME-42-e70023-s002.docx]

**Supplemental File 1**

**Case example 1:**

Person: 35 year old man with type 1 diabetes mellitus (DM1).

Background: The person has been using an insulin pump for 10 years and recently implemented a continuous glucose monitoring (CGM) system. His HbA_1c_ level is stable around 58 mmol/mol (7.5%), but he experiences fluctuations in his blood glucose levels, especially during holidays and periods of illness.

Observation Period: Over a period of 3 months, the person's CGM data is analyzed. The focus is on the trends in his estimated average glucose (GMI) and the variability in his blood glucose levels.

Week 1-4:

- GMI: 55 mmol/mol (7.2%) (average glucose: 8.9 mmol/L)
- Week 1: HbA_1c_: 58 mmol/mol (7.5%)
- Notes: The person has a stable routine and experiences little variability in his blood glucose levels.

Week 5-8:

- GMI: 62 mmol/mol (7.8%) (average glucose: 10.0 mmol/L)
- Notes: The person goes on holiday and experiences higher blood glucose levels due to changes in diet and activity.

Week 9-12:

- GMI: 57 mmol/mol (7.4%) (average glucose: 9.4 mmol/L)
- Week 9: HbA_1c_: 58 mmol/mol (7.5%)
- Notes: The person became unwell and experienced fluctuations in his blood glucose levels but recovered towards the end of the period.

Analysis:

- GMI Rate of Change: The GMI value fluctuates over the 3 months, indicating variability in the person's blood glucose management.
- HbA_1c_: Remains stable at 58 mmol/mol (7.5%), which does not reflect short-term changes.

Conclusion: By analyzing the trends in GMI over a period of time, clinicians can better understand how factors such as holidays and illness affect a person's blood glucose levels. This helps in adjusting the treatment plan to better address this variability. A single HbA_1c_ value would not have shown these fluctuations, highlighting the importance of monitoring the GMI rate of change alongside HbA_1c_.

**Case example 2**:

PISA - Person: 28 year old woman with type 1 diabetes mellitus (DM1).

Background: The person uses a continuous glucose monitoring (CGM) system to manage her blood glucose levels. She has noticed that her CGM sometimes shows low values during the night, which concerns her.

Observation Period: Over a period of 4 weeks, CGM data is analyzed to determine whether the low values at night are due to the pressure-induced sensor attenuation (PISA) phenomenon or genuine hypoglycemia.

Week 1-2:

- GMI: 54 mmol/mol (7.1%) (average glucose: 8.6 mmol/L)
- TBR (Time Below Range): 3.5%
- Notes: The person notices that her CGM often shows low values at night, especially when she sleeps on her side.

Week 3-4:

- GMI: 55 mmol/mol (7.2%) (average glucose: 8.9 mmol/L)
- TBR: 3.8%
- Notes: The person tries different sleeping positions and notices that the low values occur less frequently when she sleeps on her back.

Analysis:

- PISA Phenomenon: The low values at night seem to be related to the person’s sleeping position. When she sleeps on her side, the pressure on the sensor may cause attenuated signals and false low values.
- Genuine Hypoglycemia: To confirm whether the low values are genuine, she performs fingerstick measurements during the nighttime periods of low CGM values. These measurements show normal blood glucose levels, indicating that the low CGM values are likely due to PISA.

Conclusion: By analyzing sleeping positions and conducting additional fingerstick measurements, clinicians can determine that the low CGM values at night are likely due to the PISA phenomenon and not genuine hypoglycemia. This helps in adjusting person’s treatment plan and reducing unnecessary concerns about false low values.
