# Supplementary material for: Managing discordance between HbA1c and glucose management indicator
Source: Diabet Med. 2025 Mar 23;42(6):e70023. doi: 10.1111/dme.70023 (PMC12080991; doi:10.1111/dme.70023)
Supplement: Supplementary file 2 — Table S1. [file DME-42-e70023-s001.docx]

**Supplemental Table 1** Calibration approaches and Interference of drugs on different CGM devices

| **Sensor** | **Firm** | **Interfering Substance** | **Calibration** | **Link** |
| --- | --- | --- | --- | --- |
| FreeStyle Libre | Abbott (USA) | Ascorbic acid (vitamin C) and salicylic acid (Aspirin) | Factory calibrated, no manual calibration needed | <https://www.abbott.com/global-sites.html> |
| FreeStyle Libre2 | Abbott (USA) | Ascorbic acid (vitamin C) | Factory calibrated, no manual calibration needed | <https://www.abbott.com/global-sites.html> |
| Lingo | Abbott (USA) | Ascorbic acid (vitamin C) | Factory calibrated, no manual calibration needed | <https://www.abbott.com/global-sites.html> |
| Freestyle Libre3 | Abbott (USA) | > 1000 mg Ascorbic acid (vitamin C) per day | Factory calibrated, no manual calibration needed | <https://www.abbott.com/global-sites.html> |
| Dexcom G5 | Dexcom (USA) | Acetaminophen (Paracetamol), Hydrea (or Hydroxycabamide) | Manual calibration required, usually twice a day | <https://www.dexcom.com/global> |
| Dexcom G6 | Dexcom (USA) | Hydroxyurea (or Hydroxycabamide) | Factory calibrated, no manual calibration needed unless no sensor code is entered | <https://www.dexcom.com/global> |
| Dexcom G6 Pro | Dexcom (USA) | Hydroxyurea (or Hydroxycabamide) | Factory calibrated, no manual calibration needed. | <https://www.dexcom.com/global> |
| Dexcom ONE | Dexcom (USA) | Hydroxyurea (or Hydroxycabamide) | Factory calibrated, calibration is optional | <https://www.dexcom.com/global> |
| Dexcom G7 | Dexcom (USA) | Hydroxyurea (or Hydroxycabamide) | Factory calibrated, calibration is optional | <https://www.dexcom.com/global> |
| iPro2 Professional CGM | Medtronic (USA) | Hydrea (or Hydroxycarbamide), Acetaminophen (Paracetamol) | Manual calibration required, usually twice a day. | <https://europe.medtronic.com/xd-en/index.html> |
| Enlite Sensor | Medtronic (USA) | Hydrea (or Hydroxycarbamide), Acetaminophen (Paracetamol) | Manual calibration required, usually two to four times a day. | <https://europe.medtronic.com/xd-en/index.html> |
| Guardian Sensor 3 (MMT-7020) | Medtronic (USA) | Hydrea (or Hydroxycarbamide), Acetaminophen (Paracetamol) | Manual calibration required, usually two to four times a day. | <https://europe.medtronic.com/xd-en/index.html> |
| Guardian Sensor 4 (MM780G) | Medtronic (USA) | Hydrea (or Hydroxycarbamide), Acetaminophen (Paracetamol) | Manual calibration required, usually two to four times a day. | <https://europe.medtronic.com/xd-en/index.html> |
| Simplera Sync sensor | Medtronic (USA) | Hydrea (or Hydroxycarbamide), Acetaminophen (Paracetamol) | Factory calibrated, no manual calibration needed | <https://europe.medtronic.com/xd-en/index.html> |
| GlucoMen Day CGM | Waveform (USA) | not specified | Manual calibration required, usually once a day | <https://glucomenday.com/newplatform/en/> |
| Touchcare Slim 14 CGM | Medtrum (China) | Ascorbic acid (Vitamin C) and Acetaminophen (Paracetamol) | Manual calibration required, usually once a day | <https://www.medtrum.com/> |
| Touchcare Nano CGM | Medtrum (China) | Ascorbic acid (Vitamin C) and Acetaminophen (Paracetamol) | Factory calibrated, calibration is optional | <https://www.medtrum.com/> |
| Medtrum S9 CGM | Medtrum (China) | Ascorbic acid (Vitamin C) and Acetaminophen (Paracetamol) | With sensor code: No calibration;  No senser code: calibrate on the first day twice | <https://www.medtrum.com/> |
| (GlucoRX) AiDEX | MicroTech Medical (China) | Not specified | Factory calibrated, calibration is optional | <https://www.glucorx.co.uk/glucorx-aidex/> |
| AiDEX Lite | MicroTech Medical (China) | Not specified | Manual calibration required, usually once a day | <https://www.intuitivetherapeutics.co.nz/about/aidex-cgm> |
| Glunovo i3 | Infinovo (China) | Acetaminophen (Paracetamol) | Manual calibration required, usually twice a day. | <https://www.infinovo.com/en/Products/10.html> |
| Glunovo p3 | Infinovo (China) | Acetaminophen (Paracetamol) | Factory calibrated, no manual calibration needed | <https://www.infinovo.com/en/Products/11.html> |
| Glunovo Flash | Infinovo (China) | Acetaminophen (Paracetamol) | Factory calibrated, calibration is optional | [www.infinovo.com](http://www.infinovo.com/) |
| Sibionics GS1 CGM | Sibionics (China) | ? | Sensor code calibration | <https://www.alrt.com/> |
| iCan (TrueView) | Sinocare (China) | Not specified | ? | <https://www.sibionicscgm.com/> |
| MyGuard CT-14 | Yuwell (China) | Not specified | Manual calibration required, usually once a day | [https://www.yuwell.com/en/index.php/news/newsinfo/107.html](https://caresensair.com/en_US/) |
| Anytime CT3 | Yuwell (China) | Not specified | Sensor code calibration, calibration is optional | <https://www.yuwell.com/en/index.php/news/newsinfo/107.html> |
| MeiQi RGMS-II | MeiQi (China) | Not specified | Manual calibration required | [www.meiqi-cgm.com](http://www.meiqi-cgm.com/) |
| MeiQi RGMS-III | MeiQi (China) | Not specified | Manual calibration required | [www.meiqi-cgm.com](http://www.meiqi-cgm.com/) |
| CareSens Air | iSens (SouthKorea) | Not specified | Manual calibration required, usually once a day | <https://caresensair.com/en_US/> |
| iFree2 | Bionime (Taiwan) | ? | Manual calibration required, usually once a day | [www.bionime.com](http://www.bionime.com/) |
| CGM Eversense E3 | Senseonics (USA) | Dexamethasone, Dexamethasone acetate | Manual calibration required, usually twice a day | https://www.ascensiadiabetes.com/eversense/eversense-cgm-system/sensor/ |
|  |  | Mannitol, Sorbitol, Antibiotics of the tetracycline class |  |  |
| Eversense 365 | Sensionics (USA) | Dexamethasone, Dexamethasone acetate | Manual calibration required, usually twice a day | <https://www.ascensiadiabetes.com/eversense/eversense-cgm-system/sensor/> |
|  |  | Mannitol, Sorbitol, Antibiotics of the tetracycline class |  |  |
| SugarBEAT & ProBEAT CGM | Nemaura Medical Inc.(UK) | Not specified | Manual calibration required | <https://sugarbeat.com/home/> |
